# Quantifying the social symptoms of autism using motion capture

**DOI:** 10.1038/s41598-019-44180-9

**Published:** 2019-05-22

**Authors:** Ian Budman, Gal Meiri, Michal Ilan, Michal Faroy, Allison Langer, Doron Reboh, Analya Michaelovski, Hagit Flusser, Idan Menashe, Opher Donchin, Ilan Dinstein

**Affiliations:** 1 Psychology Department, Ben Gurion University, Beer Sheva, Israel; 2 Negev Autism Center, Ben Gurion University, Beer Sheva, Israel; 3 Pre-School Psychiatry Unit, Soroka University Medical Center, Beer Sheva, Israel; 4 Zusman Child Development Center, Soroka University Medical Center, Beer Sheva, Israel; 5 Public Health Department, Ben Gurion University, Beer Sheva, Israel; 6 Biomedical Engineering Department, Ben Gurion University, Beer Sheva, Israel; 7 Cognitive & Brain Sciences Department, Ben Gurion University, Beer Sheva, Israel

**Keywords:** Human behaviour, Diagnostic markers

## Abstract

Autism Spectrum Disorder (ASD) is a remarkably heterogeneous condition where individuals exhibit a variety of symptoms at different levels of severity. Quantifying the severity of specific symptoms is difficult, because it either requires long assessments or observations of the ASD individual, or reliance on care-giver questionnaires, which can be subjective. Here we present a new technique for objectively quantifying the severity of several core social ASD symptoms using a motion capture system installed in a clinical exam room. We present several measures of child-clinician interaction, which include the distance between them, the proportion of time that the child approached or avoided the clinician, and the direction that the child faced in relation to the clinician. Together, these measures explained ~30% of the variance in ADOS scores, when using only ~5 minute segments of “free play” from the recorded ADOS assessments. These results demonstrate the utility of motion capture for aiding researchers and clinicians in the assessment of ASD social symptoms. Further development of this technology and appropriate motion capture measures for use in kindergartens and at home is likely to yield valuable information that will aid in quantifying the initial severity of core ASD symptoms and their change over time.

## Introduction

Assessing the existence and severity of ASD symptoms in children is difficult and often requires a lengthy interview with the parents, multiple interactions with the child, and sometimes even a visit to observe the child in the kindergarten. While clinical assessments can include structured tests such as the autism diagnostic observation schedule (ADOS)^1^ and the autism diagnostic interview – revised (ADI-R)^2^, these clinical evaluations are mostly focused on determining whether a diagnosis is warranted or not, and offer relatively limited quantitative information regarding the severity of specific symptoms. For example, the DSM-5 allows clinicians to define the severity of the condition on a scale of 1–3 according to the support that the child will require^3^. Similarly, the ADOS-2 offers comparison scores that estimate the severity of the condition on a scale of 1–10^4^. These scales have a limited range and combine a variety of behaviors and symptoms into a single score. This means that ASD children with different symptoms and severities can end up with an identical score, thereby undermining the ability of existing clinical tests to quantify specific ASD symptoms and measure how they change over time, and in response to treatment.

Developing objective, quantitative, and sensitive measures for specific ASD symptoms is extremely important for several reasons. First, ASD is a remarkably heterogeneous condition in terms of its phenotype^5^, genotype^6^, and hypothesized underlying mechanisms^7^. Quantitative measures of specific ASD symptoms are, therefore, critical for identifying ASD sub-groups with distinct phenotypes. Second, determining the efficacy of existing and novel treatments requires the availability of sensitive quantitative measures that can assess change over time and determine the impact of each treatment on specific symptoms. Third, quantitative measures of specific symptoms may be useful for early detection (or risk assessment) of specific sub-types of ASD, even before the condition can be diagnosed by conventional means. Note that developing such measures for young children with ASD is likely to have the largest clinical impact given the flexibility of early development^8^ and the efficacy of early ASD interventions^9^.

Previous attempts to quantify core social symptoms in children with ASD have mostly used manual coding of live or video-taped observations, or utilized parental questionnaires. For example, studies have used observations of children in their natural habitat (i.e., kindergarten) to manually code the number and duration of social interactions^10^. Others have coded the amount of eye contact, tendency to smile, and general social activity^11^. Such studies often report that children with ASD tend to spend less time engaged in social interactions^12–14^, initiate fewer social encounters^12^, and exhibit poorer quality of social interaction^10^ when compared to typically developing children. In parallel, studies have reported that children with ASD exhibit lower scores on a variety of parent or teacher questionnaires that estimate social behaviors and preferences^15,16^. While such quantification is very useful, manual scoring and questionnaires are subjective and time consuming. This limits their reliability across sites and applicability in large-scale longitudinal studies or clinical routines.

A promising alternative for manual coding is automated coding using motion-tracking technologies, which can track the movements of multiple individuals simultaneously and quantify their interactions. Such technology has been used extensively in the study of social interactions in animals^17^, enabling large studies of fruit flies^18^ and mice^19^, including mouse models of ASD^20^. Similar approaches have been used to study human behavior in adults^21^, and even to identify repetitive movements in older children with ASD^22^ or typically developing adults mimicking such movements^23^. However, to the best of our knowledge this technology has not been used so far to study social interactions in children or to assess the severity of social symptoms in ASD children.

In the current study we utilized a marker-free motion-capture system, that integrated information from four synchronized Microsoft Kinect sensors. We analyzed the ADOS assessments of 44 children who were referred to the Negev Autism Center with a suspicion of ASD. Most of the ADOS assessment is conducted with the child and clinician seated next to a table so that the child can focus on completing specific tasks. However, every ADOS session also includes a 5–10-minute period called “free play” where the child is encouraged to leave their seat and freely explore their surroundings. This segment of the ADOS was used when quantifying the social behavior of each participating child with the motion capture system. We quantified several basic measures of social interaction, which included the distance between the child and the clinician, the relative amount of movement that the child made towards (i.e., approach) or away (i.e., avoid) from the clinician, and the amount of time that the child faced the clinician. These measures explained ~30% of the variability in ADOS scores across children. It is remarkable that quantification of the children’s movements in relation to the clinician, even from a short 5-minute segment of the ADOS, was indicative of their individual ADOS scores. We, therefore, suggest that these objective and automated measures of social-interaction can add an important quantifiable dimension to the assessment of ASD children. Furthermore, this technology can be adapted for the home and kindergarten environments, and with additional research into relevant motion tracking measures may enable truly ecological measurements of social difficulties, how they change over time, and in response to treatment.

## Methods

### Subjects

Forty-four children with a suspicion of ASD were recruited for this study. (35 boys and 9 girls, Mean age: 39 months, std: 15.2, range: 14–72 months). Children and parents were recruited by the clinical staff at the Negev Autism Center (www.negevautism.org), which is located in Soroka University Medical Center (SUMC). All children who were referred to SUMC with a suspicion of ASD after January 2015 completed an ADOS assessment and were incorporated into the center’s database^24^. The study was approved by the SUMC Ethics committee and parents of all participating toddlers provided written informed consent. All research was performed in accordance with the guidelines and regulations of the Helsinki committee.

### Experimental setup

A clinical assessment room at the Negev Autism Center was modified for motion-tracking research by installing four Kinect Cameras (Kinect V2, Microsoft inc. USA). The cameras were installed at a height of 1.5 m in each corner of the rectangular room (5.8 by 3 m), and each camera was connected to a dedicated computer. We used IPI Motion Capture (IPIsoft inc. Russia) to synchronize the recordings in time and space across the four cameras. The Kinect cameras were programed to record infrared depth images at a sampling rate of 30 fps with a resolution of 512 × 424^25^. Note that this setup used only depth images from the Kinect sensors (i.e., no video) to perform 3D motion-tracking, thereby ensuring the privacy of all participants.

### Data acquisition

Children were recorded during a clinical assessment using either the toddler’s module or modules 1–3 of the ADOS-2 test^1^, which lasted approximately 40 minutes. All of the examined assessments contained a 3–12-minute segment of “free play” where the clinician and child freely interacted in the middle of the room. This part of the assessment is meant to give the clinician an opportunity to determine whether the child initiates interactions, communicates spontaneously, explores the toys that are on the floor, plays with toys in a symbolic manner, performs stereotypical movements, etc… We focused our analyses on this segment of the ADOS, because in other segments the child is usually seated in a confined manner at a table next to the clinician. The motion tracking measures were associated with the ADOS scores from the same session.

### Pre-processing

We used IPI Mocap Studio (IPIsoft inc. Russia) to generate a 3-D cloud-point representation of the room in each movie frame. After removing the room background (the background contains static objects in the room, such as the furniture), this cloud-point representation mostly contains information about the people in the room. IPI was then used to fit a skeletal model containing 27 joints to the cloud-point data of the child and the clinician in each frame of the examined recordings. Manual examination of the skeletal fits revealed that hand and leg joints were sometimes unstable and unreliable across frames while torso and head joints were highly reliable. We, therefore, limited all further analyses to 11 torso and head joints as well as the estimated locations of the two eyes. The center of mass (CM) was calculated as the center of these 11 joints for the clinician and child separately. The child’s face direction (FD) was computed as a vector perpendicular to the head and originating between the two eye positions. We then computed the angle between the direction of this vector and the clinician’s location on each frame.

### Data analysis

**Figure 1:**
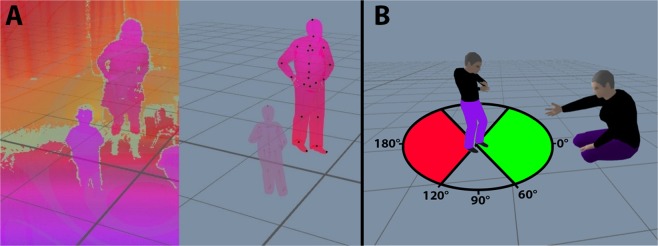
Experimental setup. (**A**) Example of a single frame as captured by a single Kinect depth camera (left) and after integrating the images from all 4 cameras, segmenting individuals from background, and fitting the skeletons to both in a 3D reference frame (right). (**B**) Schematic presentation of the approach (green) and avoid (red) directions, which were calculated as the child’s movement direction relative to the clinician.

We quantified different aspects of the child’s movements in the room throughout the extracted segment using several measures. The first was the mean distance between the child and the clinician, representing their proximity. The second was the percent of time that the child approached the clinician (i.e., moved toward the clinician ±60°, Fig. 1B). The third was the percent of time that the child avoided the clinician (i.e., moved away from the clinician ±60°, Fig. 1B). Unlike the distance measure, which was affected by the movements of the clinician, the approach and avoidance measures were solely based on the movements of the child. The fourth measure combined the approach and avoid measures by quantifying the mean movement angle of the child with respect to the clinician, weighted by the movement velocity. Children who moved towards the clinician more often than away from the clinician had angles that were less than 90° and vice versa.

$$meanMA=\frac{1}{\sum \Vert {V}_{i}\Vert }\sum _{i=1}^{N}\Vert {V}_{i}\Vert \cdot M{A}_{i}$$

Where

$$M{A}_{i}\mbox{--}{\rm{Movement}}\,{\rm{angle}}\,{\rm{on}}\,{\rm{frame}}\,{\rm{i}}\,{\rm{of}}\,{\rm{the}}\,{\rm{child}}\,{\rm{relative}}\,{\rm{to}}\,{\rm{the}}\,{\rm{clinician}}$$

$${V}_{i}\mbox{--}{\rm{Velocity}}\,{\rm{on}}\,{\rm{frame}}\,{\rm{i}}$$

$$N\mbox{--}{\rm{Number}}\,{\rm{of}}\,{\rm{frames}}$$

A final measure quantified the face direction (FD) of the child relative to the clinician’s location. We computed the average angle between a vector representing the direction of the child’s gaze and a vector representing the direction of the clinician. The gaze direction of the child was calculated from the top 4 joint locations, which represented the top and bottom of the head as well as the two eyes. Specifically, we computed a line between the top and bottom of the head. We then found a point in the middle of the two eyes. Finally, we computed the gaze vector, which was perpendicular to the line between top and bottom of the head and intersected the point between the two eyes. The angle between this gaze vector and the vector pointing in the direction of the clinician was computed for each frame and then averaged across frames to quantify the FD.

### Statistics

We computed Pearson’s correlation coefficients between each of the motion-tracking measures described above and the ADOS scores. We also performed the correlations separately for the social affect (SA) and restricted and repetitive behaviors (RRB) severity scores. The statistical significance of each correlation was determined using a randomization/permutation test. We performed 10,000 iterations where subject identities were shuffled randomly to create a null-distribution of random correlation coefficients for each motion tracking measure. Statistical significance was then established by determining whether the true correlation coefficient fell below the 2.5 or above the 97.5 percentile of this null distribution (i.e., equivalent to a two-tailed p-value of 0.05). Finally, we performed a step-wise multiple linear regression analysis where the dependent variable was the ADOS score and the independent variables were the motion-tracking measures. The tested regression models included each of the motion-tracking measures separately and together in all possible combinations. We then ranked the regression models according to their adjusted R squared values.

## Results

We examined how each of several motion tracking measures of child-clinician interaction were correlated with the total ADOS scores of the children. We also examined the social affect (SA) and restricted and repetitive behavior (RRB) scores of the ADOS separately, to assess the specific relationship between the motion tracking measures and the severity of distinct ASD symptoms.

### Mean distance between child and clinician

**Figure 2:**
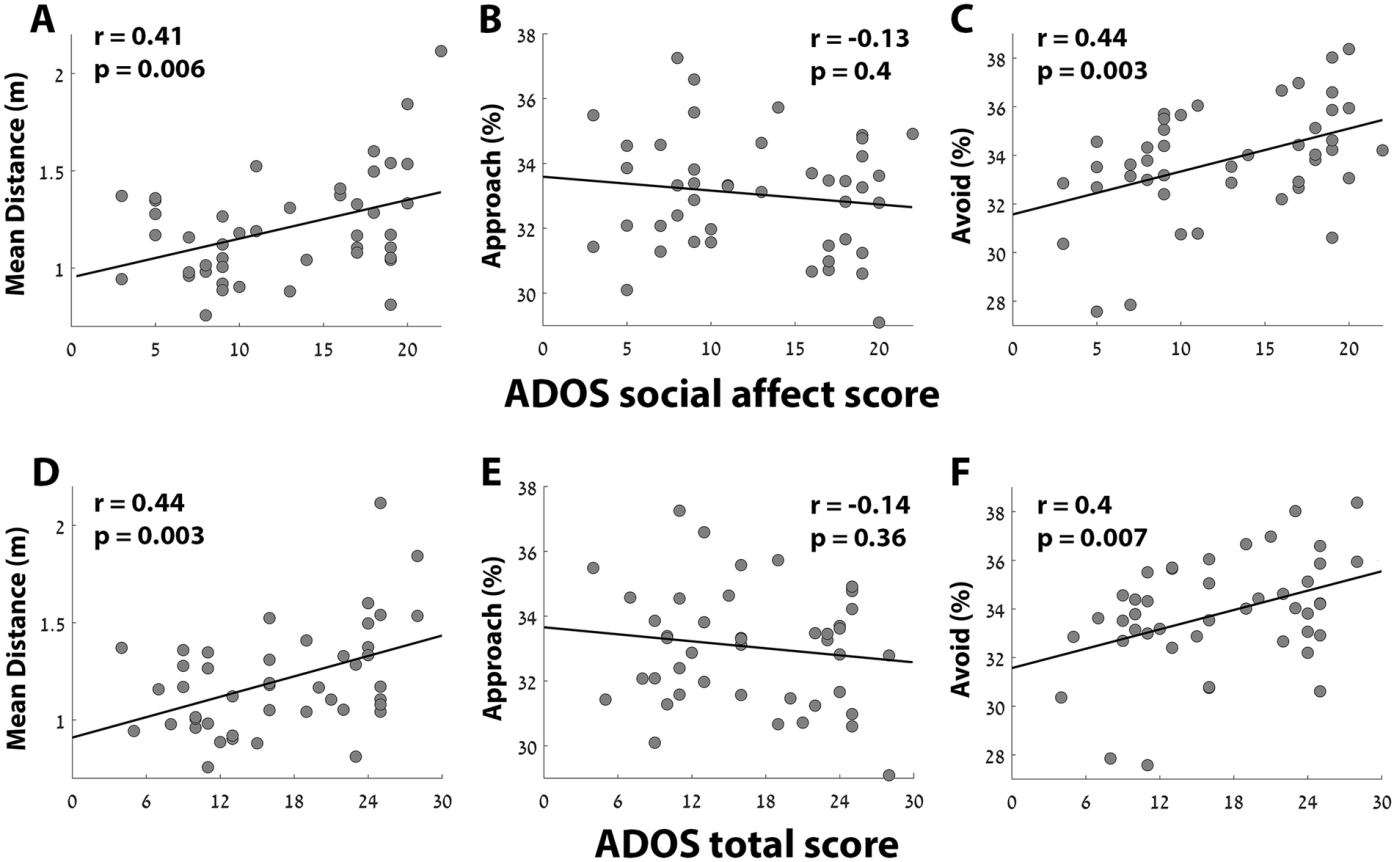
Scatter plots demonstrating the relationship between individual ADOS scores and the mean distance between clinician and child (**A,D**), the percent of time that the child approached the clinician (**B,E**), and the percent of time that the child avoided the clinician (**C,F**). Relationships with both the ADOS social affect scores (**A–C**) and total scores (**D–F**) are presented. Black line: least squares linear fit. Pearson’s correlation coefficient and statistical significance are noted in each panel.

The mean distance between the child and the clinician differed across children. While some tended to be, on average, 0.8 m from the clinician, others were >1.5 m from the clinician (Fig. 2A,D). The mean distance throughout the examined segment was positively correlated with the children’s total ADOS scores (r(44) = 0.44, p = 0.002), ADOS social affect (SA) scores (r(44) = 0.41, p = 0.003), and ADOS repetitive and restricted behaviors (RRB) scores (r(44) = 0.36, p = 0.02), demonstrating that children with more severe symptoms tended to be further away from the clinician.

### Approach and Avoidance behaviors

We then quantified the percentage of time that the child approached (moved towards) or avoided (moved away from) the clinician (Fig. 2). Individual tendency to avoid the clinician was significantly correlated with the ADOS total (r(44) = 0.4, p = 0.005) and SA (r(44) = 0.44, p = 0.002) scores, but not with the ADOS RRB scores (r(44) = 0.15, p = 0.33). A corresponding negative correlation was also apparent with individual tendencies to approach the clinician, but this relationship was not significant for ADOS total (r(44) = −0.14, p = 0.32), SA (r(44) = −0.13, p = 0.38), or RRB (r(44) = −0.1, p = 0.53) scores.

**Figure 3:**
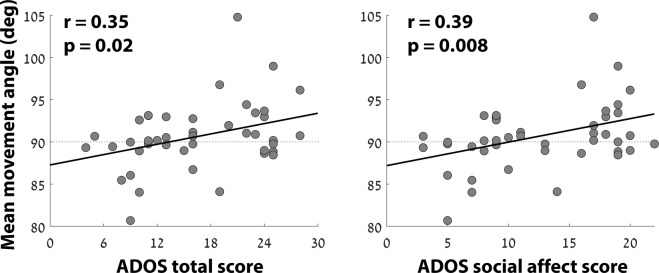
Scatter plots demonstrating the relationship between individual ADOS total scores (left) or ADOS SA scores (right) and mean movement angle of the child with respect to the clinician. Black line: least squares linear fit. Pearson’s correlation coefficient and statistical significance are noted in each panel.

We created a composite measure of the approach and avoidance behavior for each child by computing the mean movement angle of the child with respect to the clinician (Fig. 3). When moving directly towards the clinician, the angle would equal 0°, while moving away would yield an angle of 180° (Fig. 1B). The children’s mean movement angles were significantly correlated with the ADOS total (r(44) = 0.35, p = 0.02) and SA (r(44) = 0.39, p = 0.008) scores, but not with the ADOS RRB scores (r(44) = 0.11, p = 0.48). Note that the approach and avoid measurements, and their composite, exhibited considerably stronger relationships with ADOS measures of SA rather than RRB, demonstrating the specificity of these measures for quantifying the severity of social symptoms.

### Face direction of the child

**Figure 4:**
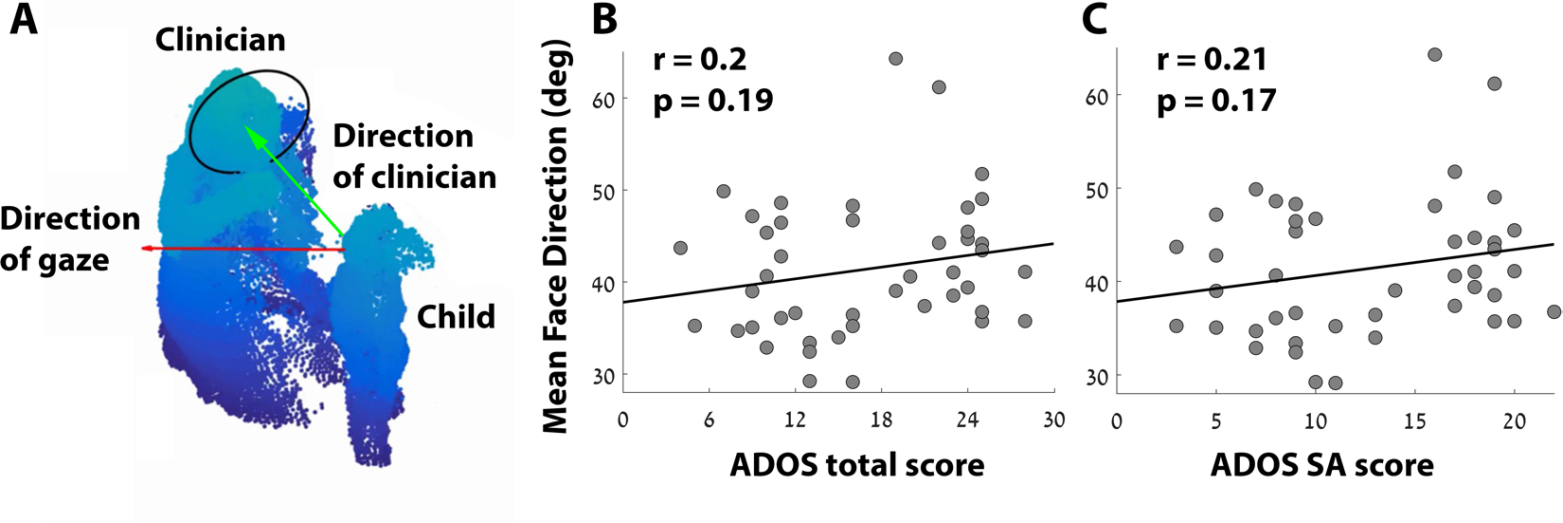
Child’s facing direction relative to the clinician (FD). A single frame of a cloud-point display demonstrating the computation of the angle between the child’s direction of gaze (red vector) and the direction of the clinician (green vector) (**A**). Scatter plots demonstrating the relationship between individual ADOS total scores (**B**) or SA scores (**C**) and mean FD. Black line: least squares linear fit. Pearson’s correlation coefficient and statistical significance are noted in each panel.

We examined the relationship between ADOS scores and the mean direction that the child was facing relative to the clinician. We quantified the average angle that the child was facing relative to the clinician, the larger the angle the further away the child was facing (Fig. 4A). There was a positive relationship between face direction (FD) angle and ADOS scores indicating that children with more severe ASD symptoms tended to face further away from the clinician. This relationship, however, was not statistically significant for either the ADOS total (r(44) = 0.2, p = 0.19), SA (r(44) = 0.21, p = 0.17), or RRB (r(44) = 0.08, p = 0.59) scores (Fig. 4). Note that the FD measure also exhibited a relatively stronger relationship with ADOS SA scores than RRB scores.

### Combining the measures

The results above revealed that the two motion tracking measures with the strongest relationship to ADOS SA scores were distance and %avoid, each explaining ~19% of the variance in ADOS SA scores (Fig. 2). To determine the potential combined power of the different measures described above, we performed a step-wise multiple regression analysis (see methods). We compared the ability of different regression models to explain the ADOS SA scores of the individual children. The model with the highest adjusted r^2^ value (adj. r^2^ = 0.29) included the distance, avoid %, and movement angle measures (Table 1). Note, however, that there were a variety of models with very similar adjusted r^2^ values, indicating that there was a lot of overlap in the explanatory power of the different variables. For example, dropping the movement angle measure from the regression model had a minor effect on the adjusted r^2^ (adj. r^2^ = 0.28).

**Table 1:** Top five multiple regression models with the best ability to explain individual differences in either the ADOS total scores or the ADOS SA scores. The motion-tracking measures (i.e., independent variables) included in each model are listed along with the F-stat, p-value, r^2^, and adjusted r^2^ of each model.

Dependent variable: ADOS total				
Independent variables	F-stat	p-value	r^2^	Adj. r^2^
distance, avoid %	9.37	>0.001	0.31	0.28
distance, avoid %, FD	6.63	>0.001	0.32	0.27
distance, avoid %, movement angle	6.73	>0.001	0.32	0.27
distance, avoid %, FD, movement angle	5.2	0.002	0.33	0.27
distance, avoid %, approach %	6.1	0.002	0.31	0.26
**Dependent variable: ADOS SA**				
distance, avoid %, movement angle	6.73	>0.001	0.34	0.29
distance, avoid %, FD	6.63	>0.001	0.33	0.28
distance, movement angle, FD, approach %	5.19	0.002	0.35	0.28
distance, avoid %	9.37	>0.001	0.31	0.28
distance, avoid %, movement angle, approach %	4.93	0.003	0.34	0.27

## Discussion

A common defining characteristic of ASD children is their tendency to avoid social interactions. The results of the current study demonstrate that this characteristic can be quantified in an objective, automated manner by applying motion tracking techniques in the clinical assessment room. ASD children with higher ADOS scores (more severe symptoms) tended to avoid interaction and kept a greater distance from the clinician (Figs 2–4). These significant correlations between the ADOS scores and motion tracking measures seem particularly promising given that we examined only a short ~5 min segment of the ADOS where the child was freely interacting with the clinician. Note that during the rest of the ADOS the child and clinician are purposefully seated next to a table, which does not allow the child freedom to move towards/away from the clinician. Extracting the same measures in other settings, where the child is free to move around for longer periods of time while they interact with clinicians, parents, or peers, is likely to improve the quantification of social difficulties in ASD children. Note that the measures presented in this study were more strongly correlated with ADOS SA scores rather than ADOS RRB scores. This demonstrates that specific motion tracking measures can be used to quantify particular ASD symptoms.

### Quantifying social symptoms in ASD

The most widely used standardized clinical assessment for quantifying ASD symptoms is the ADOS^1^, which is a structured diagnostic tool that was developed to help confirm the ASD diagnosis according to DSM 5^3^ criteria. While the ADOS has separate subscales for SA and RRB symptoms, these scales are relatively limited in range and combine a variety of heterogeneous symptoms. For example, the SA score is the sum of individual items that measure joint attention, eye contact, initiation of social communication, reciprocal interaction, and other age related social behaviors. Each of these items is scored on a scale of 0–2, which is extremely limited in sensitivity. Hence, two children with an identical ADOS SA score may have different social symptoms at different levels of severity. Additional popular tests for ASD screening (e.g., Modified Checklist for Autism in Toddlers^26^ and Social Communication Questionnaire^27^) or diagnosis (e.g., Autism Diagnostic Interview^2^) have the same problem in that they offer limited ability to quantify specific ASD symptoms.

Quantification techniques for measuring the severity of specific social symptoms have been reported in numerous studies using manual coding of behavioral observations^12^. For example, it has been reported that ASD children exhibit significantly fewer spontaneous initiations of social interactions^28,29^, fewer approach behaviors during parent-child interactions^30^, spend less time playing and interacting with peers^10,11,31^, spend more time in solitary activities^32^, and keep larger distances from others^33,34^. While these measures are useful for quantifying the severity of specific social symptoms in a sensitive manner, manual coding techniques are extremely laborious and are, therefore, not suitable for large-scale studies or daily clinical routines. Indeed, most of the studies described above analyzed short observations from small samples of 20–30 children.

The goal of the current study was to demonstrate that motion tracking algorithms can automate some of the measures used to quantify the severity of social symptoms in young ASD children. By focusing on robust measures of distance and quantification of approach and avoidance behaviors, is was possible to explain a considerable part of the variability in ADOS scores across children. Note that the examined motion tracking measures were more strongly related to social symptoms (i.e., correlated with ADOS SA scores), rather than restricted and repetitive behaviors.

When interpreting our results, it is important to consider that context and surroundings are critical factors that may influence social behavior. Here, we quantified social behavior during the ‘free play’ segment of the ADOS assessment^1^. While this segment may differ across children and ADOS modules, the same clinician administered all of the ADOS sessions reported in the current study, thereby enhancing context reproducibility. Quantifying social behaviors using motion tracking in multiple contexts would be extremely useful for assessing the strengths and weaknesses of individual children and for assessing improvements in specific settings.

### Quantifying social interactions in animal models of ASD

In contrast to contemporary behavioral research with ASD children, which is heavily reliant on manual coding techniques, behavioral research with ASD animal models relies heavily on objective, automated behavioral tracking techniques^35^. Most interesting are ecological motion tracking techniques that can identify multiple individual animals in a social setting and track their behavior relative to each other^17^. Such systems have been used, for example, to characterize the structure of social interactions in fruit flies^18^ and large-scale group social dynamics of mice^19^. A recent study with BTBR mice, a popular ASD mouse model^36^, used similar motion tracking techniques to demonstrate that BTBR mice remain further away from control mice and exhibit reduced social exploration behaviors^20^.

A variety of new commercially available technologies for tracking human behaviors are emerging^37^. While several studies have reported that similar motion tracking techniques can identify postural problems^38^ and certain repetitive behaviors^22^ in children with ASD, to the best of our knowledge, this is the first study to implement measures of social interactions involving the relative movements of multiple individuals using motion tracking. The ability to quantify social symptoms in young ASD children in this manner will enable further research to relate specific findings across human and animal studies. Furthermore, combining additional automated measures from other behavioral domains such as speech (e.g., human speechome project^39^) may offer additional utility in quantifying the development of social symptoms.

### Choice of motion tracking system

The motion tracking system described in this paper, utilizing 4 synchronized Kinect cameras, is somewhat excessive for quantifying most of the reported measures. We initially built this system with the goal of studying motor control in children with ASD. However, we failed in our attempts to acquire stable and accurate limb positions that would have enabled us to quantify movement kinematics in the children. We believe that this was due to three main challenges. The first is that the Kinect cameras and associated software were designed to extract adult movements, where the larger size of the limbs makes it easier to identify and track their location in space. The second is that the Kinect system assumes that individual subjects are standing in front of the camera without touching one another. ASD children, however, often sit and even lie down on the floor during ADOS assessments, and sometimes make physical contact with the clinician. These situations violate the assumptions of the Kinect algorithms and cause errors in the identification of joint locations. The third is that the sampling rate of the Kinect cameras, 30 frames per second, is too low to accurately capture the kinematic properties of children’s movements.

For these reasons we focused our analyses on gross measures of movement, which mostly relied on extracting the center of mass of the child and the clinician using the joint locations of the torso and head (i.e., excluding all limb joints, see Methods). Given the promising utility of these gross measures of movement for quantifying the social symptoms of ASD, future efforts could extract these measures using simpler and cheaper technologies (e.g., single camera from above or attaching a positional marker to each individual). Simplifying the data acquisition setup would be an important step in creating a system that could be implemented in ecological settings such as at home or in the kindergarten. The current explosion of commercially available digital devices for continuous telemetry of children are likely to greatly facilitate these efforts^37^.

## Conclusions

One of the biggest problems in contemporary ASD research is the shortage of sensitive and objective measures for assessing longitudinal changes in behavioral symptoms. The development of automated behavioral tracking techniques that can directly quantify the behavior of children (i.e., “digital phenotyping”) is, therefore, of great importance for both basic research and clinical assessment of treatment efficacy. Similar systems have been of great value in large-scale studies of social behavior in a variety of animal models. This study demonstrates the utility of several gross measures of child-clinician interactions for quantifying social ASD symptoms. The ability of these motion-tracking measures to explain ~30% of the variability in ADOS scores from only a ~5-minute recording of the ADOS test, demonstrates their great potential utility for both scientific research and clinical practice. Finally, adapting such systems to the home and kindergarten settings and performing further research to validate the appropriate motion tracking measures for these settings, will enable quantification of social behavior in the children’s natural environments, which are rarely examined in ASD research.

## Data Availability

Raw data will be made available upon reasonable request from the authors.

## References

[1] Lord, C. *et al*. The Autism Diagnostic Observation Schedule-Generic: A standard measure of social and communication deficits associated with the spectrum of autism. *J. Autism Dev. Disord*, 10.1023/A:1005592401947 (2000). 11055457

[2] Lord, C., Rutter, M. & Le Couteur, A. Autism Diagnostic Interview-Revised: A revised version of a diagnostic interview for caregivers of individuals with possible pervasive developmental disorders. *J. Autism Dev. Disord*, 10.1007/BF02172145 (1994). 10.1007/BF02172145 7814313

[3] American Psychiatric Association & Association, A. P. *Diagnostic and Statistical Manual of Mental Disorders. Arlington*, 10.1176/appi.books.9780890425596.744053 (American Psychiatric Publishing, 2013).

[4] Esler, A. N. *et al*. The Autism Diagnostic Observation Schedule, Toddler Module: Standardized Severity Scores. *J. Autism Dev. Disord*, 10.1007/s10803-015-2432-7 (2015). 10.1007/s10803-015-2432-7 PMC4898775 25832801

[5] Happé F, Ronald A, Plomin R (2006). Time to give up on a single explanation for autism. Nat. Neurosci..

[6] Jeste SS, Geschwind DH (2014). Disentangling the heterogeneity of autism spectrum disorder through genetic findings. Nat. Rev. Neurol..

[7] State MW (2012). Neuroscience. The emerging biology of autism spectrum disorders. Science.

[8] Hensch TK (2005). Critical period plasticity in local cortical circuits. Nat. Rev. Neurosci..

[9] Zwaigenbaum L (2015). Early Intervention for Children With Autism Spectrum Disorder Under 3 Years of Age: Recommendations for Practice and Research. Pediatrics.

[10] Lord, C. & Magill-Evans, J. Peer interactions of autistic children and adolescents. *Dev. Psychopathol*, 10.1017/S095457940000674X (1995).

[11] Bauminger, N., Shulman, C. & Agam, G. Peer Interaction and Loneliness in High-Functioning Children with Autism. *J. Autism Dev. Disord*, 10.1023/A:1025827427901 (2003). 10.1023/a:1025827427901 14594329

[12] Kennedy, C. H. & Shukla, S. Social Interaction Research for People with Autism as a Set of Past, Current, and Emerging Propositions. *Behav. Disord* (1995).

[13] Sigman, M. *et al*. Continuity and change in the social competence of children with autism, Down syndrome, and developmental delays. *Monogr. Soc. Res. Child Dev*, 10.2307/3181510 (1999). 10.1111/1540-5834.00002 10412222

[14] McGee, G. G., Feldman, R. S. & Morrier, M. J. Benchmarks of social treatment for children with autism. *Journal of Autism and Developmental Disorders*, 10.1023/A:1025849220209 (1997). 10.1023/a:1025849220209 9261663

[15] Posserud, M. B., Lundervold, A. J. & Gillberg, C. Autistic features in a total population of 7–9-year-old children assessed by the ASSQ (Autism Spectrum Screening Questionnaire). *J. Child Psychol. Psychiatry Allied Discip*, 10.1111/j.1469-7610.2005.01462.x (2006). 10.1111/j.1469-7610.2005.01462.x 16423148

[16] Constantino JN (2003). Validation of a Brief Quantitative Measure of Autistic Traits: Comparison of the Social Responsiveness Scale with the Autism Diagnostic Interview-Revised. J. Autism Dev. Disord..

[17] Robie, A. A., Seagraves, K. M., Egnor, S. E. R. & Branson, K. Machine vision methods for analyzing social interactions. *J. Exp. Biol*, 10.1242/jeb.142281 (2017). 10.1242/jeb.142281 28057825

[18] Schneider, J., Dickinson, M. H. & Levine, J. D. Social structures depend on innate determinants and chemosensory processing in Drosophila. *Proc. Natl. Acad. Sci*, 10.1073/pnas.1121252109 (2012). 10.1073/pnas.1121252109 PMC3477376 22802679

[19] Shemesh, Y. *et al*. Correction: High-order social interactions in groups of mice. *Elife*, 10.7554/eLife.03602 (2014). 10.7554/eLife.03602 PMC4052486 24920500

[20] Hong W (2015). Automated measurement of mouse social behaviors using depth sensing, video tracking, and machine learning. Proc. Natl. Acad. Sci. USA.

[21] Borges, P. V. K., Conci, N. & Cavallaro, A. Video-based human behavior understanding: A survey. *IEEE Trans. Circuits Syst. Video Technol*, 10.1109/TCSVT.2013.2270402 (2013).

[22] Gonçalves, N., Costa, S., Rodrigues, J. & Soares, F. Detection of stereotyped hand flapping movements in Autistic children using the Kinect sensor: A case study. In *2014 IEEE International Conference on Autonomous Robot Systems and Competitions, ICARSC 2014*, 10.1109/ICARSC.2014.6849788 (2014).

[23] Kang, J. Y. *et al*. Automated tracking and quantification of autistic behavioral symptoms using microsoft kinect. In *Studies in Health Technology and Informatics*, 10.3233/978-1-61499-625-5-167 (2016). 27046572

[24] Meiri G (2017). Brief Report: The Negev Hospital-University-Based (HUB) Autism Database. J. Autism Dev. Disord..

[25] Berger, K. *et al*. Markerless Motion Capture using multiple Color-Depth Sensors. *Vision, Model. Vis. 2011*, 10.2312/PE/VMV/VMV11/317-324 (2011).

[26] Robins, D. L., Fein, D., Barton, M. L. & Green, J. A. The Modified Checklist for Autism in Toddlers: An Initial Study Investigating the Early Detection of Autism and Pervasive Developmental Disorders. *J. Autism Dev. Disord*, 10.1023/A:1010738829569 (2001). 10.1023/a:1010738829569 11450812

[27] Chandler, S. *et al*. Validation of the Social Communication Questionnaire in a population cohort of children with autism spectrum disorders. *J. Am. Acad. Child Adolesc. Psychiatry*, 10.1097/chi.0b013e31812f7d8d (2007). 10.1097/chi.0b013e31812f7d8d 17885574

[28] Bauminger-Zviely, N., Karin, E., Kimhi, Y. & Agam-Ben-Artzi, G. Spontaneous peer conversation in preschoolers with high-functioning autism spectrum disorder versus typical development. *J. Child Psychol. Psychiatry Allied Discip*, 10.1111/jcpp.12158 (2014). 10.1111/jcpp.12158 24304222

[29] Duffy, C. & Healy, O. Spontaneous communication in autism spectrum disorder: A review of topographies and interventions. *Research in Autism Spectrum Disorders*, 10.1016/j.rasd.2010.12.005 (2011).

[30] Doussard-Roosevelt, J. A., Joe, C. M., Bazhenova, O. V. & Porges, S. W. Mother-child interaction in autistic and nonautistic children: Characteristics of maternal approach behaviors and child social responses. *Dev. Psychopathol*, 10.1017/S0954579403000154 (2003). 10.1017/s0954579403000154 12931828

[31] Wong, C. & Kasari, C. Play and joint attention of children with autism in the preschool special education classroom. *J. Autism Dev. Disord*, 10.1007/s10803-012-1467-2 (2012). 10.1007/s10803-012-1467-2 PMC4205103 22350340

[32] Locke, J., Shih, W., Kretzmann, M. & Kasari, C. Examining playground engagement between elementary school children with and without autism spectrum disorder. *Autism*, 10.1177/1362361315599468 (2016). 10.1177/1362361315599468 PMC4779076 26341991

[33] Gessaroli, E., Santelli, E., di Pellegrino, G. & Frassinetti, F. Personal Space Regulation in Childhood Autism Spectrum Disorders. *PLoS One*, 10.1371/journal.pone.0074959 (2013). 10.1371/journal.pone.0074959 PMC3781155 24086410

[34] Candini, M. *et al*. Personal space regulation in childhood autism: Effects of social interaction and person’s perspective. *Autism Res*, 10.1002/aur.1637 (2017). 10.1002/aur.1637 27157094

[35] Silverman, J. L., Yang, M., Lord, C. & Crawley, J. N. Behavioural phenotyping assays for mouse models of autism. *Nature Reviews Neuroscience*, 10.1038/nrn2851 (2010). 10.1038/nrn2851 PMC3087436 20559336

[36] McFarlane HG (2008). Autism-like behavioral phenotypes in BTBR T+tf/J mice. Genes, Brain Behav..

[37] Baker, J. T., Germine, L. T., Ressler, K. J., Rauch, S. L. & Carlezon, W. A. Digital devices and continuous telemetry: opportunities for aligning psychiatry and neuroscience. *Neuropsychopharmacology*, 10.1038/s41386-018-0172-z (2018). 10.1038/s41386-018-0172-z PMC6224592 30120409

[38] Dawson G (2018). Atypical postural control can be detected via computer vision analysis in toddlers with autism spectrum disorder. Sci. Rep..

[39] Meylan SC, Frank MC, Roy BC, Levy R (2017). The Emergence of an Abstract Grammatical Category in Children’s Early Speech. Psychol. Sci..

